# Optimal Usability Test Procedure Generation for Medical Devices

**DOI:** 10.3390/healthcare11030296

**Published:** 2023-01-18

**Authors:** Jeehoon Shin, Hyuk Lee

**Affiliations:** 1 College of Informatics, Korea University, Seoul 02841, Republic of Korea; 2 Department of Computer Engineering, Changwon National University, Changwon 51140, Republic of Korea

**Keywords:** usability test, medical device, validation, test procedure, testing cost

## Abstract

Medical device usability testing offers many benefits, including finding medical device usage errors and providing safety to users. As usability testing becomes mandatory for medical devices, manufacturers are increasing the cost burden. In order to perform a high-quality usability test, it is important to implement a usability test procedure, but guidelines for this are lacking. In this paper, we propose a method to systematically design and implement a usability test procedure. We propose methods to reduce test time-costs and apply them to implement the final procedure. Next, by applying the proposed method to sinus surgical navigation system, it is shown that the total time was reduced by 21% compared to the usability summative test procedure previously used in the same system.

## 1. Introduction

A medical device is a product used for diagnosing, treating, alleviating, monitoring or preventing a disease. Although the functions and performance of medical devices are gradually developing, the usage and interface of the medical devices are becoming more complicated. In particular, the interface of a medical device embedded with software is more complex. A complicated interface in a medical device causes use errors. These use errors can directly or indirectly harm patients or users. Between 2012 and 2015 in the U.S., there were 423 recalls (more than 140 per year) related to interface errors and 499 identified software interface errors [1]. Another study suggests that injuries due to use errors of medical devices are significantly higher than those caused by device errors [2]. Design-induced errors in medical devices can lead to patient injuries and deaths. Usability testing aims to reduce these issues and the resulting use errors [3].

IEC 62366 [4] mandates usability testing as a requirement for medical device approval. These tests confirm whether a use error occurs while using the device and whether the risk due to the use error is within an acceptable range. In usability testing, it is not possible to test all procedures due to time and cost reasons. Therefore, in order to perform meaningful usability tests, it is important to design the test procedures focusing on high-risk tasks based on risk analysis.

However, there are some practical difficulties in applying usability testing. The first is the lack of proper guidelines for developing usability validation procedures [5], and the second is the burden on small manufacturers for the time and cost required to perform the validation procedures. Therefore, a systematic method for designing a cost-effective usability validation procedure is required.

In this paper, we propose a method to design and implement a time-cost effective test procedure for a summative usability validation test [6] by selecting the lowest time-cost scenario. To do this, we first develop an extended uFMEA-based activity diagram which includes additional information about task criticality, dependency and time-cost. The diagram is then converted to an abstracted graph, removing unnecessary nodes from the graph, and we identify all possible paths except those that violate dependency. Finally, the path with the lowest time-cost among all paths is selected as the final usability test procedure.

The contributions of this paper are as follows: (1) a systematic method for deriving usability validation test procedures is presented, (2) a way to implement cost-effective procedures for medical device manufacturers is proposed, and (3) it is verified by applying the proposed method to actual medical devices.

This paper is organized in the following manner. In Section 2, current research medical device usability is reviewed. In Section 3.1, we describe preliminary hazard analysis for medical devices, provide a task analysis in a risk management process for usability testing and examine the system model using an activity diagram. In Section 3.2, we present a method for representing time-cost and activity dependencies. In Section 3.3, we describe how to integrate the above process to build a uFMEA-based activity diagram. Then, in Section 3.4, we propose a method to implement a usability test procedure using the uFMEA-based activity diagram. In Section 4, we apply the proposed method to sinus surgery navigation system and examine the results. In Section 5, we provide procedure generalizations and limitations. Then, we present the conclusions of this paper and discuss future research.

## 2. Related Work

There are many studies that suggest problems and solutions on various topics related to medical device usability [7,8,9,10,11,12,13,14,15,16,17,18,19,20,21,22,23]. According to several medical device usability studies, current ventilator usability is poor, the operation error rate is high, and the task completion time is long [24,25,26,27,28]. This can have serious consequences in an emergency situation. Various studies have been conducted on home ventilators [29,30], infusion pumps [31,32], insulin pumps [33] and dialysis machines [34,35] to address usability-related safety and design problems [36]. Although the quality of usability testing is highly dependent on the relevant usability test procedure, there are few guidelines on how to develop usability validation procedures for medical devices [5]. Since this can be a problem directly related to the safety of users or patients, systematic procedure development for usability tests is very important [37]. There are many cases in which usability and interface have been improved and effectively verified by applying usability testing to medical devices [12,38]. In particular, the cognitive-walkthrough method was identified by Bligard and Osvalder and Liljegren et al. [39] as an effective way to evaluate usability. However, the method has problems with a poor high-level perspective, insufficient categorization of detected usability issues and difficulty in synthesizing analysis results, so Lars-Ola Bligård et al. [3] proposed a new enhanced cognitive walkthrough (ECW). ECW is a proactive analysis method for analyzing potential usability issues [40]. Zhang et al. [41] modified the existing heuristic evaluation method for evaluating software usability, applied it to medical devices, and used it to evaluate patient safety of the device through identification and evaluation of usability problems. Cognitive walkthrough and heuristic methods are good for usability formative tests but cannot be applied as simulation-based usability summative tests.

## 3. Derivation of Usability Test Procedure Based on Risk Analysis

**Figure 1:**
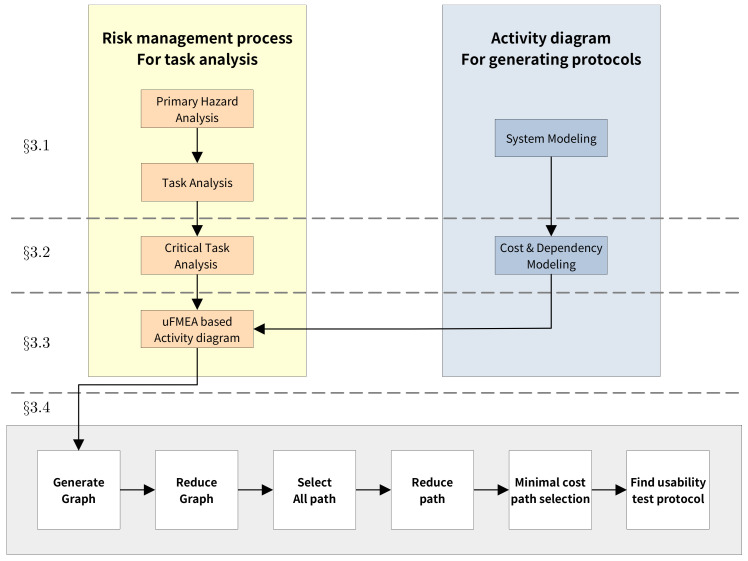
Overall process for deriving a time-cost effective usability test procedure.

In this Section, we propose a methodology for conceptualizing and designing a minimum time-cost usability test procedure for medical devices. The overall process of the proposed approach is shown in Figure 1.

Our approach consists mainly of two parts. The first part is from §3.1 to §3.3 in Figure 1, which develops an extended uFMEA-based activity diagram that includes additional information. §3.1 is a prerequisite process for developing the extended uFMEA-based activity diagram. In this process, we describe hazards and tasks associated with the user interface and a system model. Each part of this process is described in Section 3.1. §3.2 is a process for deriving additional information such as task criticality, time-cost and activity dependency. The steps for deriving this additional information are described in Section 3.2; § 3.3 details the process for developing an extended uFMEA-based activity diagram that includes additional information. Based on the information obtained from a preliminary hazard analysis, task analysis and system modelling, the criticality of each task is decided, the task is added to the system activity diagram, and the time-cost and dependency between each activity are defined to develop the extended uFMEA-based activity diagram. In Section 3.3, we describe how to develop the extended uFMEA-based activity diagrams with additional information.

In § 3.4 in Figure 1, after conceptualizing and implementing the extended uFMEA-based activity diagram as a graph, the paths are searched and the time-cost of each path is calculated to design the final usability procedure. First, by converting the developed diagram into a graph, unnecessary nodes are deleted, and all paths are implemented with the modified graph. Then, we verify whether all critical tasks are included, and if they are not, we create additional routes containing the critical tasks that were not originally included. Finally, by calculating the time-cost of each procedure, the procedure with the least time-cost is selected as the final usability procedure. In Section 3.4, we describe how to implement a final usability test procedure using an extended uFMEA-based activity diagram.

### 3.1. Prerequisites for Usability Test Procedure Derivation

#### 3.1.1. Preliminary Hazard Analysis

Preliminary Hazard Analysis (PHA) is an inductive analysis method to identify hazards, sequences of events, hazardous situations, and harm. Identified hazards are ranked on a scale of 1 to 5, respectively, according to severity and occurrence. All potential hazards and accidental events that may lead to an accident are identified. Then, identified accidental events according to their severity are ranked, and necessary risk controls and follow-up activities are identified [42]. Through this process, a risk matrix is built, and the risk is calculated according to the severity and occurrence of each hazard.

#### 3.1.2. Task Analysis

In the context of usability engineering in medical devices, a task is defined as “One or more user inter-activities with a medical device to achieve a desired result” [4]. Through task analysis, all tasks that occur in the use of medical devices are identified, and the order relationship between tasks is analyzed. Task analysis is used to find out about the interactivity between a user and a medical device to achieve a desired outcome. The results of a task analysis can take the form of a narrative, a table, or a flowchart, the latter two being the most common.

#### 3.1.3. System Model Using Activity Diagram

Activity diagrams represent the behavior of a system consisting of one or more subsystems. Activity diagrams represent the flow of control from start to finish in that system, showing the various decision paths that exist during the execution of an activity.

### 3.2. Additional Steps to Complete uFMEA-Based Activity Diagram

#### 3.2.1. Derivation Critical Task for Usability Test

According to the FDA’s Center for Devices and Radiological Health(CDRH), the Centers for Drug Evaluation and Research(CDER), and Biologics Evaluation and Research(CBER) critical tasks in the context of medical devices are defined as, “A user task which, if performed incorrectly or not performed at all, would or could cause serious harm to the patient or user, where harm is defined to include compromised medical care” [43].

**Figure 2:**
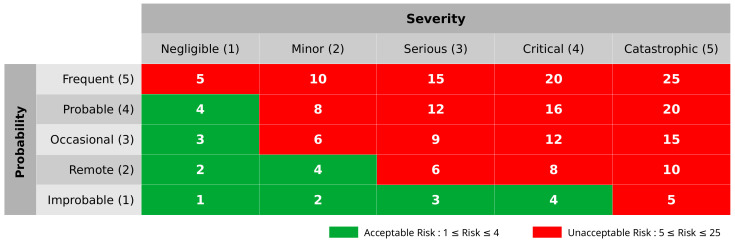
Risk matrix.

It is determined based on the risk obtained by hazard analysis for each task and classifies tasks above predefined risk as critical tasks. (e.g., task with a risk of five or higher) In order to calculate the risk, the PHA stage analyzes the task from PCA perspective and decides the severity and probability(occurrence) of hazard for each task. PCA stands for perception, cognition, and action. Then, the risk is evaluated based on the risk matrix shown in Figure 2.

**Figure 3:**
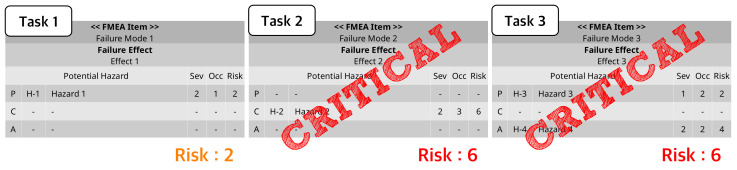
Critical task analysis.

In Figure 3, FMEA item means the contents of the task failure, and the failure effect means the expected effect in case of failure. In perception(P), cognition(C), and action(A) items, hazards and hazard IDs related to the task are listed, the severity and occurrece of each hazard are written, and each risk is calculated based on this. The sum of each risk is calculated as the total risk of the task.

In Task 3, the respective risks are two and four, and the final risk is calculated as six, which is the sum of two and four. Based on the risk matrix, tasks that exceed an acceptable level of risk, which is four, are considered critical tasks.

#### 3.2.2. Time-Cost Assessment and Activity Dependency

The time-cost of the activity represents the time required to complete the activity, and the timeCost function converts this time-cost into an integer value between 1 and 10. Within a set of tasks, 1 is assigned to the task that requires the least time, and 10 is assigned to the task that requires the most time.

(1) $$timeCost\left(t_{a}\right)=\left\{\begin{array}{cc} \left(\frac{10-1}{t_{max}-t_{min}}\times \left(t_{a}-t_{min}\right)\right)+1 & \;\mathrm{if}\;t_{max}\neq t_{min}\\ \; & \\ \;1 & \;\mathrm{otherwise} \end{array}\right.$$

The time-cost of an activity *a*, that is timeCost($t_{a}$), is calculated with the time of the most time-consuming activity $t_{max}$ and the least time-consuming activity $t_{min}$. To convert activity time to a time-cost value between 1 and 10, the ratio is calculated by dividing the difference between 1 and 10 by the difference between the minimum and maximum activity time. Multiplying the calculated ratio by the difference between the activity time and the minimum time yields the activity a time as a value between 0 and 9. We can derive a time-cost between 1 and 10 by adding 1 to the above calculation. If the minimum and maximum activity times are the same, that is, if all activities have the same time, then the time-cost of all activities is one.

For example, consider activities $a_{1}$, $a_{2}$, $a_{3}$, and $a_{4}$ which take 10, 370, 90, and 170 s to complete, respectively. Then, the timeCost of activities $a_{1}$, $a_{2}$, $a_{3}$, and $a_{4}$ are 1, 10, 3, and 5. The cost of time obtained in this way is later used to select the final usability test procedure.

Activity dependency indicates a dependency between two activities. For example, if activities $a_{1}$ and $a_{2}$ have an activity dependency, when activity $a_{1}$ is executed, $a_{2}$ must be executed before termination. In the activity diagram, it is represented by a dotted line between the activities.

### 3.3. uFMEA-Based Activity Diagram

With the hazard list, tasks, critical tasks, time-cost, and activity dependency calculated above, a uFMEA-based activity diagram is created. The uFMEA-based activity diagram is developed through the following steps.

(1) Add tasks in user interface as subsystem:
  The tasks derived from task analysis are modeled on the activity diagram. At this time, the subsystem user interface and tasks identified in the user interface are added to the diagram, and the relationship between tasks or between tasks and activities in the subsystem is shown according to the execution order.
(2) Critical task representation:
  Among the tasks, the critical tasks are colored in red, and other general tasks are colored in blue. This is displayed on the upper right of the task, and the risk of the task is indicated.
(3) Time-cost representation:
  It shows the time-cost value calculated for each activity. This is displayed at the bottom right of the activities.
(4) Dependency represent representation:
  The defined activity dependency relationship is specified in the activity diagram. Activities with activity dependency are indicated by dotted arrows.

**Figure 4:**
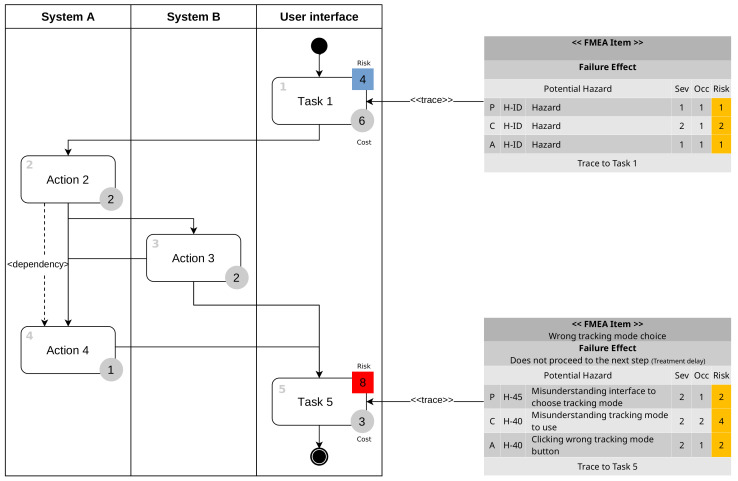
Extended uFMEA-based activity diagram.

Figure 4 is the result of performing the above four steps and shows the workflow for user interface tasks and activities of other systems. In the user interface lane, Task 1 and Task 5 are obtained through task analysis. Gray circles with numbers represent time-costs, and the colors and numbers of the squares represent the criticality of the task. In Figure 4, Task 1 has a risk level of four, so it is classified as a non-critical task and displayed in blue, and Task 5 has a risk level of eight and is classified as a critical task and displayed in red. The tables on the right side of Figure 4 show the uFMEA performed from the PCA perspective, and each uFMEA item is connected to the relevant task.

### 3.4. Derivation of Usability Test Procedures

In this paper, the usability test procedure is defined as a sequence of action and task. The usability test procedure is implemented through the following steps.

(1) Transform uFMEA-based activity diagram to graph.
(2) Delete unnecessary tasks after selecting the critical task-based initial and final task.
(3) Search all paths between the first user interface task and the last user interface task based on the deleted graph.
(4) Check the activity dependency in all searched paths, and delete the path that violates the dependency.
(5) Check the critical tasks that are not included in the remaining paths, and calculate the sum of cost of the remaining paths after creating additional critical tasks.
(6) Select the path with the lowest sum of cost as the final usability test procedure.

#### 3.4.1. Transforming uFMEA-Based Activity Diagram to Graph

The written uFMEA-based activity diagram is expressed as a graph by abstraction. Each task in the diagram is a node in the graph, and the flow of control between nodes is transformed into an edge. At this time, the activity dependency of uFMEA is not converted.

**Figure 5:**
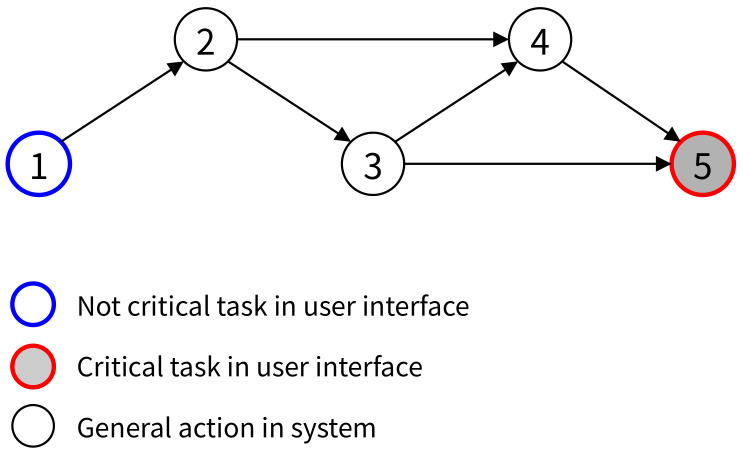
Transforming uFMEA to graph.

Figure 5 is an abstract graph of Figure 4. The action 2, 3, 4 of Figure 4 are described by Node 2, 3, 4 and Tasks 1 and 5 are converted to Node 1 and 5 with information about whether they are critical tasks or not. The flows between actions and tasks of Figure 4 are converted to edges between nodes.

#### 3.4.2. Remove Unnecessary Nodes

This step removes meaningless sequential paths. That is, unnecessary steps are reduced by removing the sequence of non-critical tasks from the initial or final task. However, if a critical task exists in the path, the path is not removed, and the critical task is set as an initial or final task.

Algorithm 1 works as follows. TaskGroup is a set of tasks, and $Node_{1}$ and $Node_{2}$ are the first and second elements of the TaskGroup, and $Node_{n}$ and $Node_{n-1}$ are the last and last n-1 elements of the TaskGroup. The initial node setting algorithm applies $Node_{1}$ and $Node_{2}$ to the algorithm at the TaskGroup. If a path does not exist between $Node_{1}$ and $Node_{2}$, or if a critical task does not exist in the path even if there is a path, $Node_{1}$ is deleted from the TaskGroup and $Node_{2}$ and $Node_{3}$ are input, and the algorithm is applied again.

**Figure 6:**
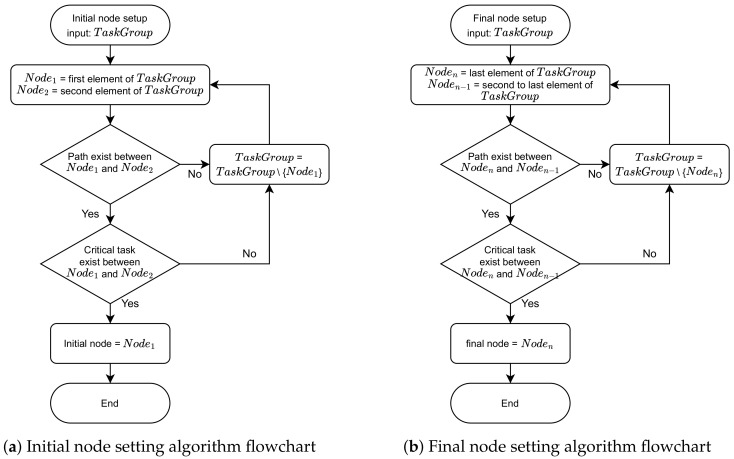
The flowchart for algorithm.

The final node setting algorithm is basically the same as the initial node setting algorithm, and the input is applied in the order of the $n^{th}$ and ${\left(n-1\right)}^{th}$TaskGroup element numbers. Figure 6 represents the initial or final node reduction flow.

**Figure 7:**
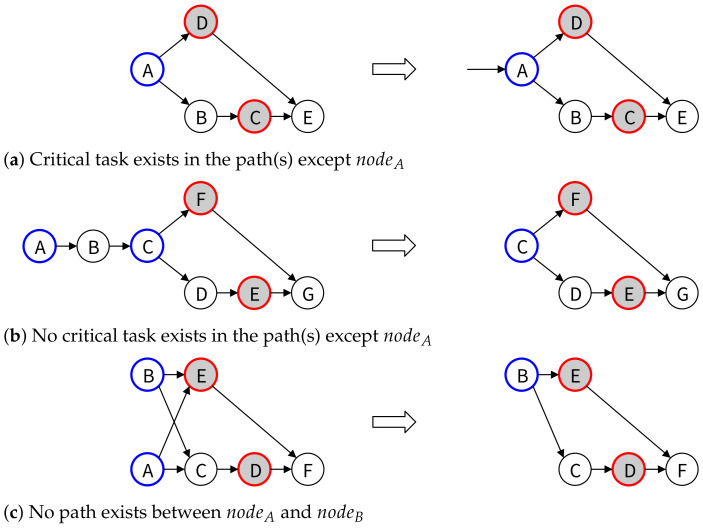
Initial node setup algorithm.

In Figure 7a, a path from $Node_{A}$ to $Node_{C}$ exists, and the path contains critical tasks, so Node A is set as the initial node. In Figure 7b, there is a path from $Node_{A}$ to $Node_{C}$, but since the path does not contain a critical task, $Node_{A}$ is deleted from the TaskGroup and applied to the algorithm again. In Figure 7c, since there is no path from $Node_{A}$ to $Node_{B}$, $Node_{A}$ is deleted from the TaskGroup and applied to the algorithm again.
**Algorithm 1:** Setting initial and final nodes.
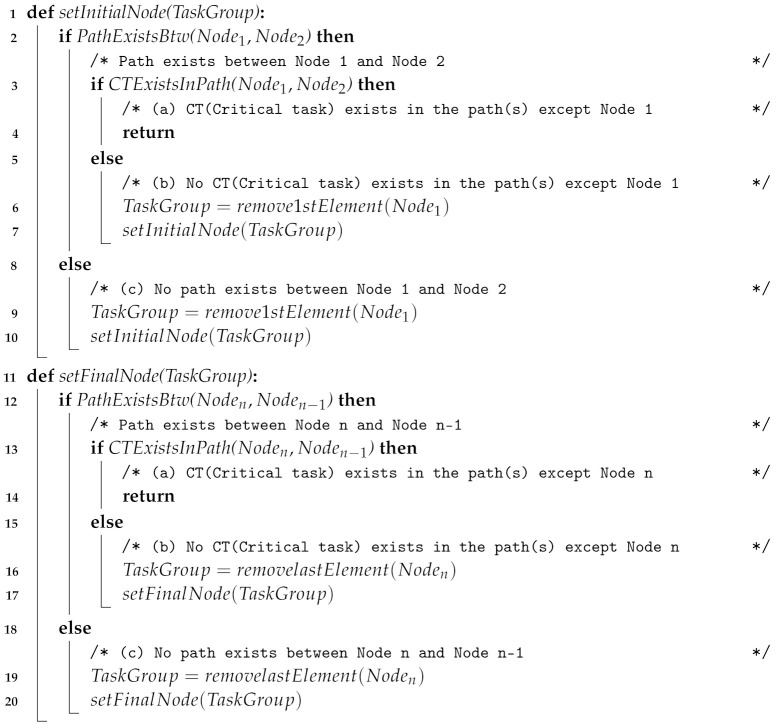


#### 3.4.3. Searching for All Paths and Removing Paths by Task Dependency

In this step, all possible paths from the first node to the last node of the TaskGroup are searched. Then, the paths that violate the dependency between tasks described in the extended uFMEA-based activity diagram among all the derived paths are removed. If Activity 2 and Activity 4 have a dependency relationship and one of the derived paths is <1, 2, 3, 5>, this path is deleted because Activity 4 is not executed after Activity 2 is executed and is terminated.

#### 3.4.4. Procedure Generation by Adding Paths to Include Remaining Critical Tasks

In this step, a procedure is created by adding a path to include all critical tasks in each path. The purpose of this paper is to design a path that performs all critical tasks. Therefore, for the derived paths, after checking the critical task that is not included in this path, it is necessary to add a path that performs the critical task. The process of adding a path is as follows.

(1) For all derived paths, check whether each path contains all critical tasks. In a path that does not include all critical tasks, the missing critical tasks are identified.
(2) Create a new path including a critical task.
  (a) Each non-included critical task implements all tasks that exist before the corresponding critical task is performed on the uFMEA-based activity diagram.
  (b) Derive all paths between each implemented task and critical task and calculate the sum of time-cost.
  (c) The path with the least sum of time-cost is selected as the additional route.

After checking critical tasks that are not included in each remaining path, add a path to include them. This path implements the paths between the tasks before the critical task and the critical task among the tasks in the TaskGroup, calculates the cost of each path, and selects the path with the lowest cost and adds it.

#### 3.4.5. Final Usability Test Procedure

In the final step, the time-cost of all paths is calculated with the time-cost of the added routes added up, and the route with the lowest time-cost is selected as the final procedure. At this time, the time-cost of each path is calculated by adding up all time-costs of one or more paths that may include all critical tasks that are not included.

## 4. Usability Test Procedure Derivation-Based Risk Analysis for Surgical Navigation

In this section, we describe a case study that applied our proposed method to a sinus surgical navigation system. Our proposed method basically works by identifying test routes and deleting unnecessary tasks among non-critical user interface tasks and tasks that violate task dependencies. For relatively simple hardware-based or firmware-based medical devices (e.g., digital blood pressure monitors, abdominal vibratory apparatus, etc.), application of the proposed method may result in little or no significant reduction in time-cost as the number of operations eliminated is small. In this respect, the sinus surgical navigation system is not too simple, and the number of tasks or test procedure paths is sufficient, making it appropriate as a case study.

**Figure 8:**
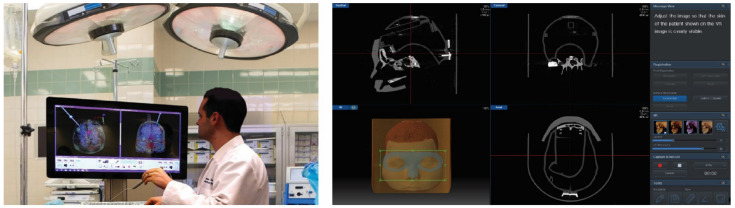
Sinus surgical navigation system.

Sinus surgical navigation systems [44] are essentially like GPS (global positioning satellite) systems for the anatomy of human nose. These systems are used to aid the surgeon in confirming the location of critical structures when the interior of the nose and sinuses is distorted by unusual anatomy or prior surgery as shown in Figure 8. There are two versions of the navigation system (magnetic system, optical system). In the magnetic system, an system control unit (SCU) and a sensor interface unit (SIU) are parts of an electromagnetic tracking system. SCU controls the electromagnetic generator, collects information from the SIUs, calculates the position and orientation of each sensor, and interfaces with the host computer. The SIU which is connected to the SCU amplifies and digitizes the signal from the sensor. This system provides functions such as augmenting the endoscope screen, screen capture, recording, and memo.

### 4.1. Prerequisites

Task analysis, risk analysis, and the system dynamics model are presented as part of the system analysis, design, and development process results. These are later used to identify critical tasks and design uFMEA-based activity diagrams. Based on each identified task, the critical task analyzes the associated risk, calculates the risk, and determines the criticality based on the risk matrix. A uFMEA-based activity diagram adds the identified tasks to a diagram that represents the performance relationship of the system and references the execution sequence between tasks to define their relationship to the functioning of the system. In addition, the uFMEA-based activity diagram is completed by specifying the time-cost and dependency relationships of each function and task.

#### 4.1.1. Preliminary Hazard Analysis

In this step, the hazard list is identified, and the results of the risk analysis related to the user interface of the surgical navigation system are displayed. Hazards and hazard ID were derived based on the hazards list in ISO 14971 [45], and new hazard factors not included in the standard can be newly defined. Table 1 shows only hazards related to the user interface of the surgical navigation system from the PHA. In the subsequent task analysis, risk is calculated by considering the severity and probability of the occurrence of hazards for each user interface task. In addition, it is possible to add new hazards that are missing or not defined in ISO 14971 while analyzing the task.

The risk matrix in the surgical navigation system derived from the hazard analysis is shown in Figure 2. The system classifies acceptability based on risk five. Then, we rank risks according to the severity and occurrence of hazards. We then classify the risks according to their severity and occurrence. These results will be used as a basis for determining critical tasks in the next step.

#### 4.1.2. Task Analysis

In the process from the start to the end of sinus surgical navigation system, all tasks interacting with the user are identified, and a sequence between the tasks is derived. A total of 10 tasks are described in Table 2.

The identified tasks and their relationships are specified together with the system dynamic model in the next step to design a uFMEA-based activity diagram.

### 4.2. Additional Steps to Complete uFMEA Based Activity Diagram

#### 4.2.1. Critical Tasks Derivation for Sinus Surgical Navigation System

**Figure 9:**
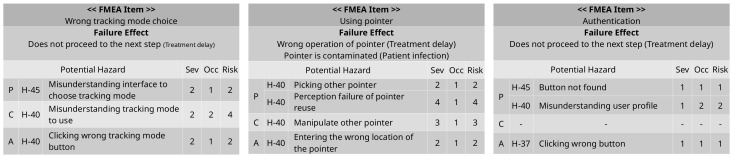
Risk analysis for sinus surgical navigation system.

Figure 9 is the uFMEA for each task from the perspective of PCA. Total risk is determined as the sum of the risks of all relevant hazards.

**Figure 10:**
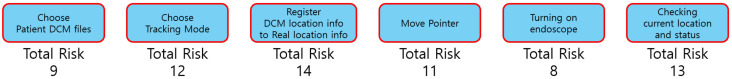
Identification of critical tasks in risk analysis.

In the navigation system, Risk 5 or higher is considered as an unacceptable risk. That is, tasks with Risk 5 or higher are identified as critical tasks. As shown in Figure 10, ’Choose Patient DCM file’, ’Choose Tracking Mode’, ’Register DCM location info to Real location info’, ’Move Pointer’, ’Turning on endoscope’, ’Checking current location and status’ tasks are considered final critical tasks.

#### 4.2.2. Time-Cost and Dependency for Sinus Surgical Navigation System

The actual time required for each task and activity was measured to obtain the time-cost of the sinus surgical navigation system. The actual time required was recorded by performing the task a total of four times and rounding to five units. The conversion values from 1 to 10 were obtained by applying the required time to (1), and the time-cost of each task and activity is shown in the Table 3.

The magnetic and optical systems transmit the position of the pointer to the navigation system upon initialization after operation. One of the two systems is selected and executed according to the purpose. Therefore, in a magnetic system, activity ’Communicate with SCU and SIU’ and activity ’Send location info(magnetic)’ have a dependent relationship, and in an optical system, activity ’Operate Optical Camera’ and activity ’Send location info(optical)’ also have a dependent relationship.

### 4.3. uFMEA-Based Activity Diagram

**Figure 11:**
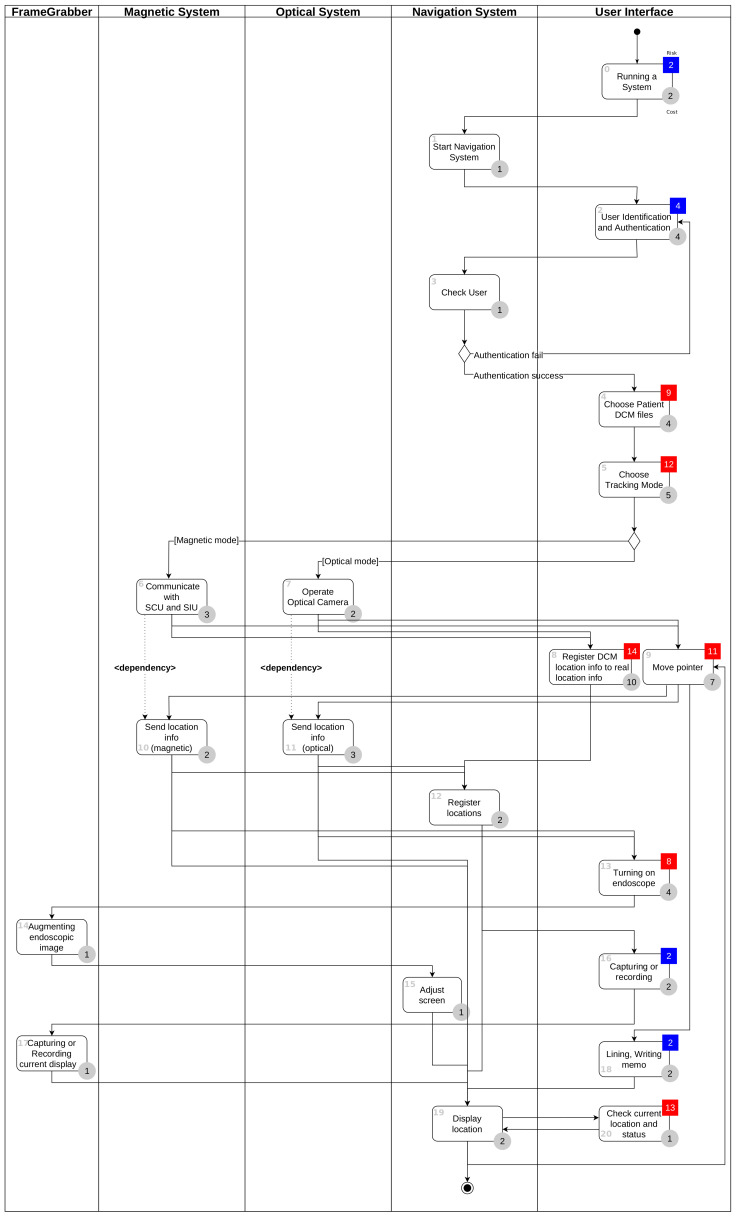
The uFMEA-based activity diagram for sinus surgical navigation system.

Figure 11 is a uFMEA-based diagram showing each task (including user interface-related tasks) and the sequence and relationship between them in the operation of sinus surgical navigation system. The system consists of a navigation system in charge of performing navigation functions, an optical camera system that tracks each location, an electromagnetic tracking system (magnetic system), a system in charge of endoscopic image processing (FrameGrabber), and the user interface responsible for user interactivity. Dependencies are represented by arrows between tasks and activities.

### 4.4. Usability Test Procedure Derivation for Sinus Surgical Navigation System

#### 4.4.1. Transform Activity Diagram to Graph

**Figure 12:**
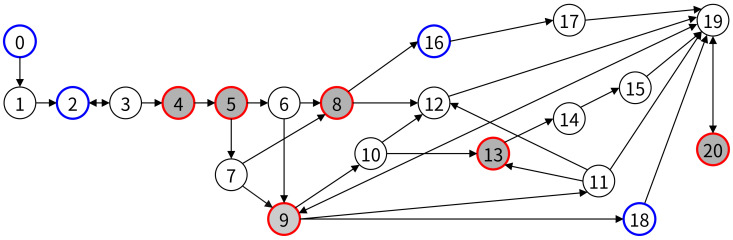
Graph for sinus surgical navigation system.

Figure 12 is a transformation of the extended activity diagram shown in Figure 11. All nodes in the graph correspond to all activities in the extended activity diagram (system activities and user interface activities), and arrows representing workflow between all activities are represented by edges between nodes. When activities are converted to nodes, the criticality is reflected, but the dependency is not.

#### 4.4.2. Remove Unnecessary Nodes

**Figure 13:**
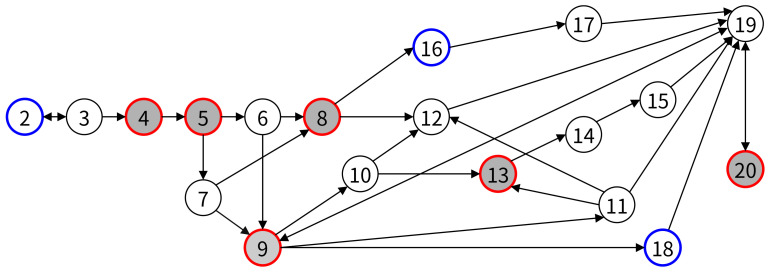
Reduced graph for sinus surgical navigation system.

Node 0, the first user interface task, has a pass to Node 2, which is the next user interface task, but is excluded from the initial node because there are no critical tasks and branches in the path. In Node 2, the path to the next user interface task, Node 4, exists, and since the critical task (Node 4) exists, we set Node 2 as the initial Node. A path exists between Node 20, the last user interface task, and Node 18, the previous user interface task, and set Node 20 as a final node because Node 20 is a critical task. Figure 13 is a graph with unnecessary nodes removed. Node 0 and Node 1 in Figure 12 are removed in a graph by Algorithm 1.

#### 4.4.3. Searching for All Paths and Removing Paths by Activity Dependency

In the reduced graph in Figure 13, we find all paths between Node 2, the first task (the first element of the TaskGroup), and Node 20, the last task. In the graph in Figure 13, there are a total of 16 paths from Node 2 to Node 20, as shown in Table 4.

Node 10 (Send location info (magnet)) is dependent on Node 6 (Communicate with SCU and SIU), and Node 11 (Send location info (optical)) has a dependency on Node 7(Operate Optical Camera) in sinus surgical navigation system. That is, when Node 6 precedes the path, Node 10 follows, and when Node 7 precedes, Node 11 must follow. Paths that violate this rule are Path 1, Path 5, Path 6, Path 7, Path 8, Path 9, Path 10, Path 11, Path 12, and Path 16 and are deleted from Table 4.

Of the 16 paths, 10 paths that violate dependency are deleted, and the remaining 6 paths become candidates for the usability test procedure.

#### 4.4.4. Procedure Generation by Adding Paths to Include Remaining Critical Tasks

Once all paths that satisfy the dependencies have been identified, we check that each path contains all critical tasks. If there is a missing critical task in a path, we add a complementary path containing the corresponding critical task. Looking at Path 2 in Table 4, the path does not include Critical Tasks 8 and 13. In this case, Paths <5, 7, 8> and <9, 10, 13> including Critical Task 8 and Critical Task 13 are added as complementary paths of Path 2. A set of paths containing all critical tasks becomes a test procedure. As shown in Table 5, the total cost of the procedures created in this way is calculated by summing the costs defined for each task.

#### 4.4.5. Final Usability Test Procedure

**Figure 14:**
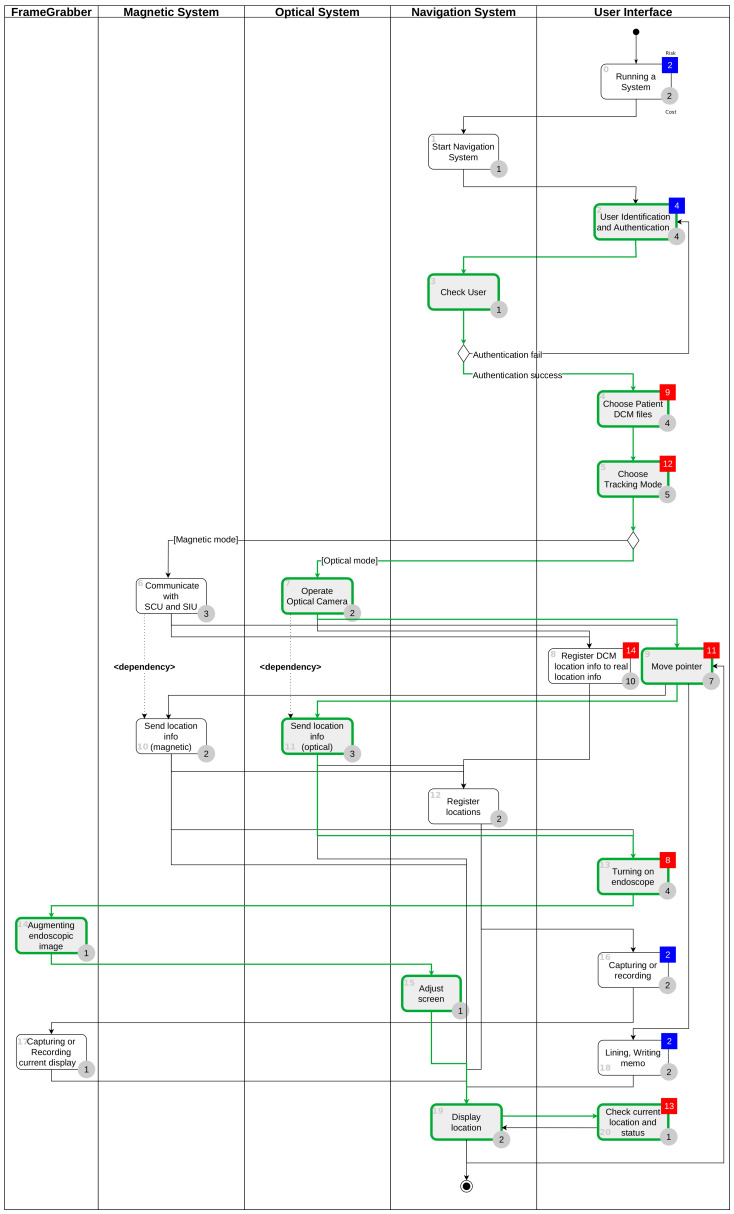
Final procedure.

**Figure 15:**
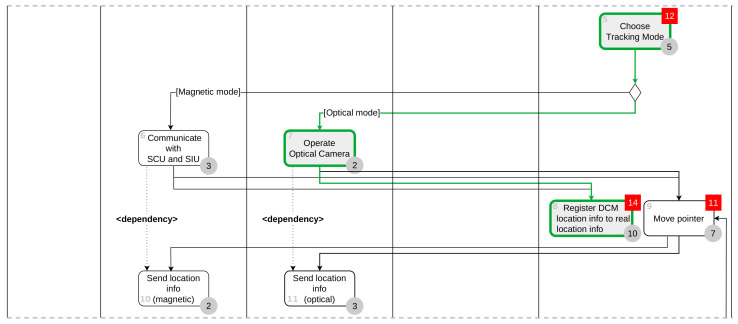
Final procedure 2.

The least expensive of Procedures 2 and 6 in Table 5 is chosen as the final usability test procedure. In this paper, Procedure 2 was chosen as the final procedure.The final usability test procedure of the final Path 1 is shown in Figure 14 and Figure 15. The original procedure was used as a usability test procedure when certifying an actual medical device, and is designed to perform all important tasks in one procedure without removing the initial and final nodes.

Activities performed in the reduced procedure by the proposed method were reduced by about 32%, and the control flow decreased by about 43% compared to the original procedure. Operation (the individual steps in the interactivity between the USER and the USER INTERFACE) decreased by about 43%, the number of user interface tasks decreased by 3, and the number of critical tasks was the same. The total cost of performing the procedure was reduced by about 21% as shown in Table 6.

## 5. Conclusions and Discussion

For medical device certification, medical device manufacturers must perform a usability test process in compliance with the IEC 62366 standard. However, there are difficulties in developing a usability test procedure because there is no guide, and in the case of a small medical device manufacturer, it can be a burden in terms of money and time-cost for a usability test.

We proposed a systematic procedure derivation method for a summative usability validation test. Due to the lack of a systematic method for deriving usability test procedures, we compared the method used for product certification with the proposed method for the same system and showed that the total cost was reduced by 21%.

Since the method we propose is based on the outputs (activity diagram, uFMEA, risk matrix, etc.) derived when developing actual medical device products, we believe that there will be no difficulty in applying it to other products.

Our method focuses on software-based medical devices. In the case of relatively simple hardware or firmware-based medical devices(e.g., abdominal vibratory apparatus, etc.), time-cost may not change significantly as a result. Because even if our method is applied, the number of tasks removed is few or none, and in the case of medical devices with simple functions(e.g., blood glucose monitors, digital blood pressure monitors, etc.), it may be meaningless because there are only a few total use paths.

In future research, we plan to extend the cost model by considering not only time-cost but also various factors such as labor cost and test bed. In addition, we will consider automating the development of usability procedures that take uFMEA-based activity diagrams as input and design minimum cost usability test procedures.

Through this paper, manufacturers can conduct a more affordable usability test, and it is expected that the performing hospital will reduce the burden of deriving test procedures.

## Figures and Tables

**Table 1:** Hazards for sinus surgical navigation system.

Hazard		Hazard ID
∘ Operational hazard		-
Functions	(1) Inaccurate or inappropriate output or functionality	H-36
	(2) Incorrect activity	H-37
Use errors	(1) Lack of attention	H-40
	(4) Knowledge base failure	H-43
∘ Information hazard		-
Label	(1) Incomplete usage description	H-45
	(3) Complex operation method	H-51

**Table 2:** Tasks for sinus surgical navigation system.

No.	Task	Task Description
1	Running a System	System start up
2	User Identification and Authentication	User Confirmation
3	Choose Patient DCM files	Confirm surgical patient and load CT data
4	Choose Tracking Mode	Task description
5	Register DCM location info to real location info	Task description
6	Move pointer	Move the pointer to determine location
7	Turning on endoscope	Endoscope system connection
8	Capturing or recording	Screen capture and video recording
9	Lining, Writing memo	Write on-screen content
10	Checking current location and status	Check on-screen location and system status

**Table 3:** Tasks for sinus surgical navigation system.

Activity	0	1	2	3	4	5	6	7	8	9	10
time(s)	30	10	100	15	100	130	80	40	300	200	50
time-cost	2	1	4	1	4	5	3	2	10	7	2
Activity	11	12	13	14	15	16	17	18	19	20	-
time(s)	60	40	100	15	15	30	10	30	30	15	-
time-cost	3	2	4	1	1	2	1	2	2	1	-

**Table 4:** All path(total 16) in the graph.

Path Number	Nodes	Dependency
Path 1	<2,3,4,5,7,9,19,20>	*Violated*
Path 2	<2,3,4,5,7,9,11,19,20>	*Ok*
Path 3	<2,3,4,5,7,9,11,13,14,15,19,20>	*Ok*
Path 4	<2,3,4,5,7,9,11,12,19,20>	*Ok*
Path 5	<2,3,4,5,7,9,10,19,20>	*Violated*
Path 6	<2,3,4,5,7,9,10,13,14,15,19,20>	*Violated*
Path 7	<2,3,4,5,7,9,10,12,19,20>	*Violated*
Path 8	<2,3,4,5,7,8,12,19,20>	*Violated*
Path 9	<2,3,4,5,6,9,19,20>	*Violated*
Path 10	<2,3,4,5,6,9,19,20>	*Violated*
Path 11	<2,3,4,5,6,9,11,13,14,15,19,20>	*Violated*
Path 12	<2,3,4,5,6,9,11,12,19,20>	*Violated*
Path 13	<2,3,4,5,6,9,10,19,20>	*Ok*
Path 14	<2,3,4,5,6,9,10,13,14,15,19,20>	*Ok*
Path 15	<2,3,4,5,6,9,10,12,19,20>	*Ok*
Path 16	<2,3,4,5,6,8,12,19,20>	*Violated*

**Table 5:** Generated procedures and costs.

Test Procedure	Set of Paths	Costs
Procedure 1	<2,3,4,5,7,9,11,19,20>, <5,7,8>, <9,10,13>	59
Procedure 2	<2,3,4,5,7,9,11,13,14,15,19,20>, <5,7,8>	52
Procedure 3	<2,3,4,5,7,9,11,12,19,20>, <5,7,8>, <9,10,13>	61
Procedure 4	<2,3,4,5,7,9,10,19,20>, <5,7,8>, <9,10,13>	59
Procedure 5	<2,3,4,5,7,9,10,13,14,15,19,20>, <5,7,8>	52
Procedure 6	<2,3,4,5,7,9,10,12,19,20>, <5,7,8>, <9,10,13>	61

**Table 6:** Usability testing cost comparison.

	Original	Reduced
	Procedure	Procedure
Number of Total activity or Activity	22	15
Number of Total Control flow	21	12
Number of Total Operation	17	10
Number of Total User Interface Tasks	11	8
Number of Total Critical Task	7	7
Estimated Cost	66	52

## Data Availability

Data sharing is not applicable to this article.

## Abbreviations

The following abbreviations are used in this manuscript:

FMEA	Failure Mode Effectiveness Analysis
DCM	Digital imaging and Communications in Medicine
IEC	International Electrotechnical Commission
ISO	International Organization for Standardization
GPS	Global Positioning System
ECW	Enhanced Cognitive Walkthrough
PHA	Preliminary Hazard Analysis
FDA	Food and Drug Administration
CDRH	Center for Devices and Radiological Health
CDER	Center for Drug Evaluation and Research
PCA	Perception, Cognition, Action
SCU	System Control Unit
SIU	Sensor Interface Unit
